# The angiotensin‐(1‐7)/Mas receptor axis protects from endothelial cell senescence via klotho and Nrf2 activation

**DOI:** 10.1111/acel.12913

**Published:** 2019-02-17

**Authors:** Alejandra Romero, Álvaro San Hipólito‐Luengo, Laura A. Villalobos, Susana Vallejo, Inés Valencia, Patrycja Michalska, Natalia Pajuelo‐Lozano, Isabel Sánchez‐Pérez, Rafael León, José Luis Bartha, María Jesús Sanz, Jorge D. Erusalimsky, Carlos F. Sánchez‐Ferrer, Tania Romacho, Concepción Peiró

**Affiliations:** ^1^ Department of Pharmacology Faculty of Medicine Universidad Autónoma de Madrid Madrid Spain; ^2^ Instituto de Investigaciones Sanitarias IdiPAZ Madrid Spain; ^3^ Instituto Teófilo Hernando Universidad Autónoma de Madrid Madrid Spain; ^4^ Department of Biochemistry Faculty of Medicine Universidad Autónoma de Madrid Madrid Spain; ^5^ Instituto de Investigaciones Biomédicas UAM-CSIC Madrid Spain; ^6^ CIBER for Rare Diseases Valencia Spain; ^7^ Servicio de Farmacología Clínica Instituto de Investigación Sanitaria Hospital Universitario de la Princesa Madrid Spain; ^8^ Department of Obstetrics and Gynecology Faculty of Medicine Universidad Autónoma de Madrid Madrid Spain; ^9^ Department of Pharmacology Universidad de Valencia Valencia Spain; ^10^ Institute of Health Research INCLIVA University Clinic Hospital of Valencia Valencia Spain; ^11^ School of Sport and Health Sciences Cardiff Metropolitan University Cardiff UK

**Keywords:** angiotensin‐(1‐7), endothelial senescence, heme oxygenase‐1, klotho, nuclear factor (erythroid‐derived 2)‐like 2, vascular aging

## Abstract

Endothelial cell senescence is a hallmark of vascular aging that predisposes to vascular disease. We aimed to explore the capacity of the renin–angiotensin system (RAS) heptapeptide angiotensin (Ang)‐(1‐7) to counteract human endothelial cell senescence and to identify intracellular pathways mediating its potential protective action. In human umbilical vein endothelial cell (HUVEC) cultures, Ang II promoted cell senescence, as revealed by the enhancement in senescence‐associated galactosidase (SA‐β‐gal+) positive staining, total and telomeric DNA damage, adhesion molecule expression, and human mononuclear adhesion to HUVEC monolayers. By activating the G protein‐coupled receptor Mas, Ang‐(1‐7) inhibited the pro‐senescence action of Ang II, but also of a non‐RAS stressor such as the cytokine IL‐1β. Moreover, Ang‐(1‐7) enhanced endothelial klotho levels, while klotho silencing resulted in the loss of the anti‐senescence action of the heptapeptide. Indeed, both Ang‐(1‐7) and recombinant klotho activated the cytoprotective Nrf2/heme oxygenase‐1 (HO‐1) pathway. The HO‐1 inhibitor tin protoporphyrin IX prevented the anti‐senescence action evoked by Ang‐(1‐7) or recombinant klotho. Overall, the present study identifies Ang‐(1‐7) as an anti‐senescence peptide displaying its protective action beyond the RAS by consecutively activating klotho and Nrf2/HO‐1. Ang‐(1‐7) mimetic drugs may thus prove useful to prevent endothelial cell senescence and its related vascular complications.

**Abbreviations**
ACE2: angiotensin‐converting enzyme 2
Ang‐(1‐7): angiotensin‐(1‐7)
Ang: angiotensin
ARE: antioxidant‐regulated elements
BSA: bovine serum albumin
FCS: fetal calf serum
HO‐1: heme oxygenase‐1
ICAM‐1: intercellular adhesion molecule‐1
IL‐1β: interleukin‐1β
Nrf2: nuclear factor‐erythroid 2‐related factor 2
RAS: renin–angiotensin system
r‐klotho: recombinant klotho
SA-β-gal: senescence‐associated β‐galactosidase
SASP: senescence-associated secretory phenotype
Sn‐PP: tin protoporphyrin IX
TIFs: telomere dysfunction‐induced foci
TRF‐1: telomere protein telomeric repeat binding factor
VCAM‐1: vascular cell adhesion molecule‐1
γH2AX: phosphorylated histone H2AX

## 1 INTRODUCTION

Vascular aging is a complex multifaceted process displaying functional and structural alterations that ultimately favor vascular disturbances, including endothelial dysfunction and atherosclerosis (Donato, Morgan, Walker, & Lesniewski, 2015). Vascular aging is indeed a main predictor of frailty and poor cardiovascular outcomes in the elderly (Nilsson, 2008).

Endothelial cell senescence is one of the major mechanisms contributing to vascular aging (Donato et al., 2015; López‐Otín, Blasco, Partridge, Serrano, & Kroemer, 2013). Moreover, a wide variety of extracellular stressors and DNA insults, including cytokines and vasoactive peptides, may promote premature senescence in endothelial cells, which consequently undergo a series of functional and morphological changes that ultimately lead to growth arrest and the acquisition of a senescence‐associated secretory phenotype (SASP) (Erusalimsky, 2009). The SASP is considered a main driver of sterile age‐related inflammation which favors leukocyte recruitment and predisposes to vascular disease (Donato et al., 2015; Tchkonia, Zhu, Deursen, Campisi, & Kirkland, 2013). In this context, the search of therapeutic tools to attenuate premature endothelial senescence arises as a promising strategy to attenuate vascular aging and its complications (Childs, Li, & Deursen, 2018).

The renin–angiotensin system (RAS) plays a pivotal role in the regulation of cardiovascular homeostasis, in both health and disease. In recent years, a number of studies have been dedicated to decipher the biological actions of novel RAS components, such as angiotensin (Ang)‐(1‐7). This heptapeptide is generated not only from angiotensin II (Ang II) through the action of angiotensin‐converting enzyme 2 (ACE2), but also from angiotensin I (Ang I) via neutral endopeptidase activity (Passos‐Silva, Brandan, & Santos, 2015). Ang‐(1‐7) is a ligand for the G‐protein‐coupled receptor Mas, the first member of the Mas‐related G‐protein‐coupled (MrgD) receptor family (Kostenis et al., 2005). Mas can be found in different extents in most organs and tissues, and such ubiquitous Mas expression relies, at least in part, on its endothelial expression in vessels from different organs, suggesting a relevant role of this receptor in the function of the endothelium (Bader, Alenina, Andrade‐Navarro, & Santos, 2014).

In the cardiovascular system, Ang‐(1‐7) has been regarded as a physiological antagonist of Ang II by opposing its vasoconstrictor, proliferative, hypertrophic, or pro‐inflammatory actions (Machado‐Silva, Passos‐Silva, Santos, & Sinisterra, 2016; Peiró et al., 2016; Simões E Silva & Teixeira, 2016; Villalobos et al., 2016). In recent studies, Ang II has been shown to directly promote endothelial cell senescence, thus contributing to vascular disease by a novel mechanism (Hsu, Lin, Lin, & Juo, 2018; Li et al., 2014). However, whether Ang‐(1‐7) may antagonize the endothelial senescence promoted by Ang II or even by other non‐RAS stressors remains to be determined.

Klotho is a protein playing a key regulatory role in bone and mineral metabolism. It is expressed at high levels not only in the kidney, but also in other tissues including the vasculature (Hu, Kuro‐o, & Moe, 2014). In recent years, klotho has been recognized as an anti‐aging protein, since the *klotho* gene inactivation displays a premature aging phenotype in mice (Kuro‐o et al., 1997). The impact of klotho on vascular function remains poorly understood, but the protein seems relevant for vascular homeostasis, since mice with defective *klotho *gene exhibit endothelial dysfunction (Saito et al., 1998).

The nuclear factor‐erythroid 2‐related factor 2 (Nrf2)/antioxidant‐regulated element (ARE) system is a major evolutionary conserved cytoprotective system. It is also nowadays considered a powerful modulator of species longevity (Loboda, Damulewicz, Pyza, Jozkowicz, & Dulak, 2016). Nrf2 responds by promoting the expression of genes encoding for anti‐oxidant and anti‐inflammatory proteins. Among them, heme oxygenase (HO)‐1 provides cell protection by degrading the pro‐oxidant heme and ultimately forming bilirubin together with the signaling gas carbon monoxide (Loboda et al., 2016).

In this study, we aimed to explore the capacity of the Ang‐(1‐7)/Mas receptor axis to antagonize cell senescence induced by Ang II and by other non‐RAS stressors. The participation of klotho and Nrf2/HO‐1 in the protective actions of Ang‐(1‐7) on endothelial cells was also analyzed.

## 2 RESULTS

### 2.1 Ang‐(1‐7) mitigates endothelial cell senescence induced by Ang II

**Figure 1:**
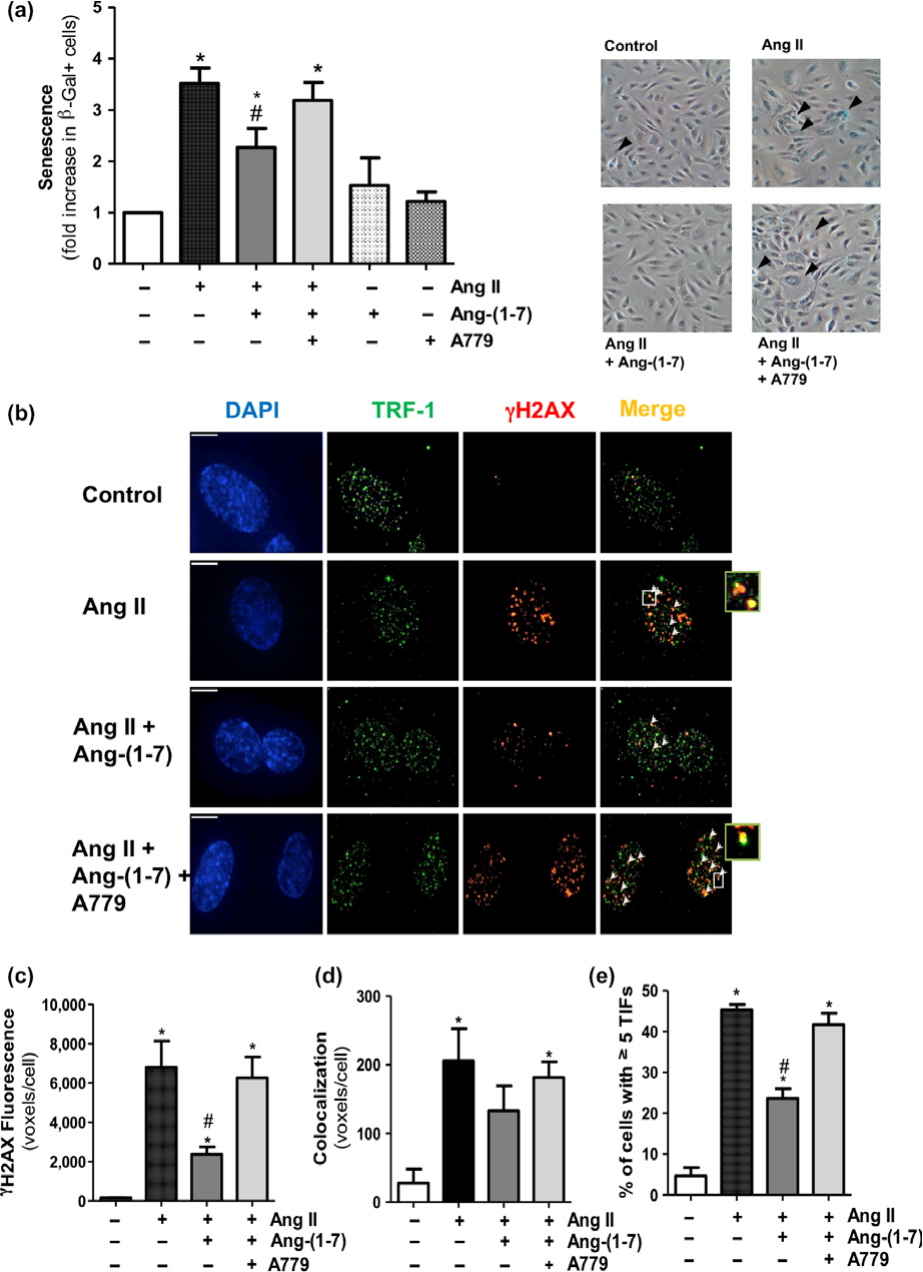
Ang‐(1‐7) counteracts endothelial cell senescence and DNA damage induced by Ang II. (a) SA‐β‐gal+ cells were quantified by manual scoring in HUVEC treated for 18 h with Ang II (100 nM), either alone or in the presence of Ang‐(1‐7) (100 nM). In some experiments, the Mas receptor antagonist A779 (1 μM) was added. Representative phase‐contrast images are shown on the right, with SA‐β‐gal+ cells (blue staining) indicated with arrowheads (200×). (b) Representative deconvolved images of HUVEC treated as described above. DNA damage foci and telomere dysfunction‐induced *foci* (TIFs) were detected by immunofluorescence microscopy with specific antibodies against γH2AX (red) and TRF‐1 (green). Cell nuclei were counterstained with DAPI (blue). Arrowheads point to sites of γH2AX and TRF‐1 colocalization (yellow). The right small boxes are enlarged views of representative merged images where colocalization of γH2AX with TRF‐1 was observed, especially in cultures treated with Ang II or IL‐1β alone or in combination with Ang‐(1‐7) + A779. Bar = 15 μm. (c) γH2AX foci and (d) TIFs were quantified as mean fluorescent voxels per cell. (e) Percentage of cells with ≥5 TIFs per cell. *p < 0.05 vs. control untreated cells, ^#^ *p* < 0.05 vs. Ang II; *n* = 3–4

The fraction of senescence‐associated β‐galactosidase (SA‐β‐gal) cells, used as a marker of cell senescence, was of 2.97 ± 0.23% in control unstimulated human umbilical vein endothelial cell (HUVEC) pre‐senescent cultures. After the exposure to Ang II (100 nM) for 18 hr, the fraction of cells positively stained for SA‐β‐gal was significantly enhanced, and this effect was mitigated by Ang‐(1‐7) (100 nM; Figure 1a). The anti‐senescence action of Ang‐(1‐7) was blunted by the selective and competitive Mas antagonist peptide D‐Ala7‐Ang‐(1‐7) (A779; 1 µM) (Figure 1a). Ang‐(1‐7) and A779 had no effect by themselves on the fraction of SA‐β‐gal+ cells (Figure 1a).

DNA damage is one major event triggering pro‐senescence responses, which results in the accumulation of phosphorylated histone H2AX (γH2AX) at the sites of injury (Erusalimsky, 2009). Consistent with this notion, Ang II also increased the number of γH2AX foci scattered over total nuclear DNA (Figure 1b,c). Moreover, Ang II augmented the co‐localization of γH2AX with the telomere protein telomeric repeat binding factor (TRF)‐1 in the so‐called telomere dysfunction‐induced foci (TIFs) (Figure 1b,d), as well as the number of endothelial cells expressing at least five TIFs (Figure 1e). Ang‐(1‐7) attenuated the impact of Ang II on DNA damage by a mechanism that was also blunted by A779 (Figure 1b–e).

We also assessed the action of Ang‐(1‐7) in late‐passage (P12) senescent HUVEC, in which the proliferation rate was markedly reduced and the average SA‐βgal+ staining in basal unstimulated conditions was shifted up to 53.52 ± 3.44% (Supporting Information Figure S1). This basal SA‐β‐gal+ staining was further increased by Ang II, but only by 1.20 ± 0.06‐fold (Supporting Information Figure S2a). In these senescent cells, Ang‐(1‐7) prevented not only the effect of Ang II, but also part of the enhanced basal SA‐β‐gal+ staining (Supporting Information Figure S2a).

### 2.2 Ang‐(1‐7) protects from endothelial cell senescence induced by non‐RAS stressors

**Figure 2:**
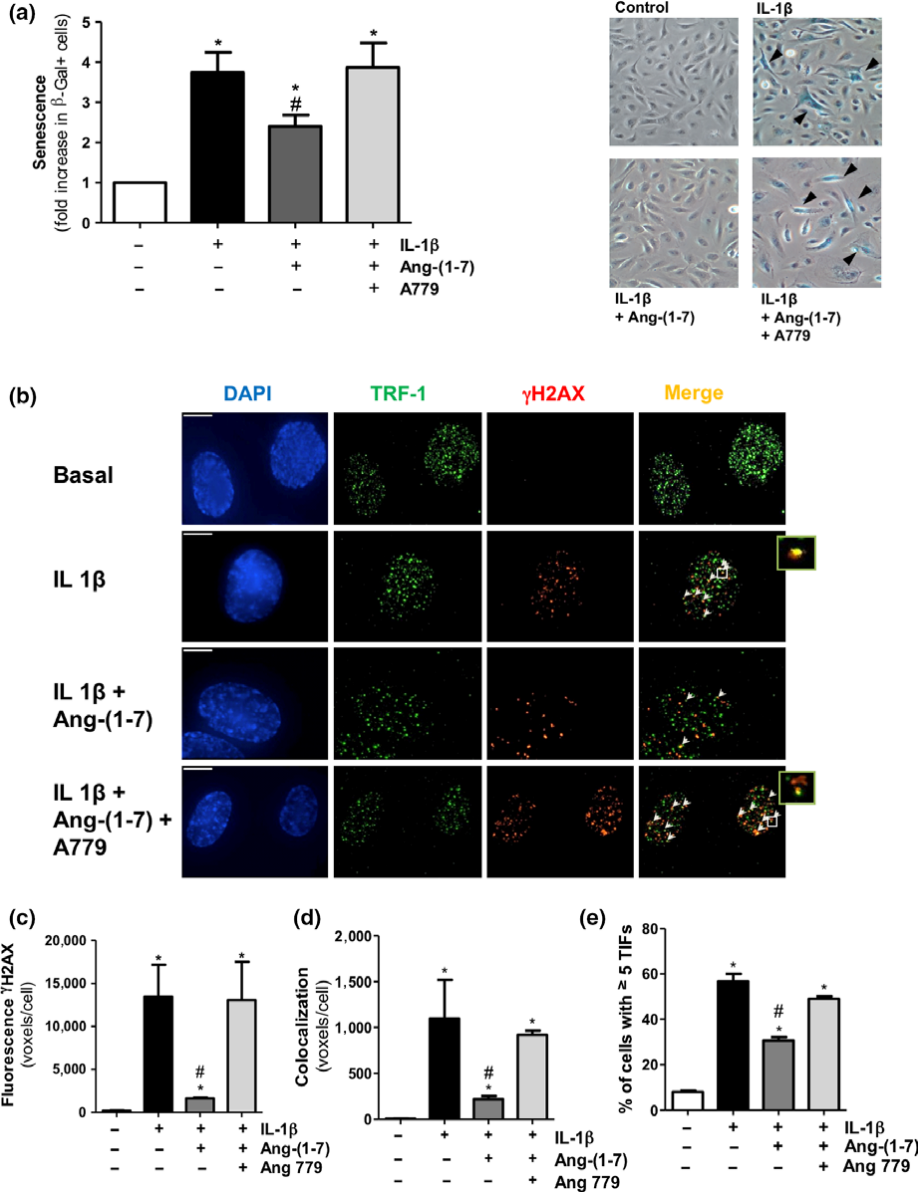
Ang‐(1‐7) counteracts endothelial cell senescence and DNA damage induced by the non‐RAS stressor IL‐1β. (a) SA‐β‐gal+ cells were quantified by manual scoring in HUVEC treated for 18 h with IL‐1β (2.5 ng/ml), either alone or in the presence of Ang‐(1‐7) (100 nM) with or without the Mas receptor antagonist A779 (1 μM). Representative phase‐contrast images are shown on the right, with SA‐β‐gal+ cells (blue staining) indicated with arrowheads (200×). (b) Representative deconvolved images of HUVEC treated as described above. DNA damage foci and telomere dysfunction‐induced foci (TIFs) were detected by immunofluorescence microscopy with specific antibodies against γH2AX (red) and TRF‐1 (green). Cell nuclei were counterstained with DAPI (blue). Arrowheads point to sites of γH2AX and TRF‐1 colocalization (yellow). The right small boxes are enlarged views of the merged images where colocalization of γH2AX with the telomere protein telomeric repeat binding factor (TRF)‐1 was observed in the so‐called telomere dysfunction‐induced foci (TIFs). Bar = 15 μm. (c) γH2AX foci and (d) TIFs were quantified as mean fluorescent voxels per cell. (e) Percentage of cells with ≥5 TIFs per cell. *n* = 3–7; *p < 0.05 vs. control untreated cells, ^#^ *p* < 0.05 vs. IL‐1β

Ang‐(1‐7) has been proposed as a physiological antagonist of Ang II. However, we next aimed to determine the capacity of the heptapeptide to counteract endothelial senescence induced by other stressors independent of the RAS. The pro‐inflammatory cytokine interleukin (IL)‐1β was chosen based on its growing relevance in human vascular disease (Libby, 2017; Ridker et al., 2017). Similar to Ang II, IL‐1β (2.5 ng/ml) increased SA‐β‐gal+ cell number (Figure 2a) and augmented γH2AX levels at both telomeric and non‐telomeric sites, as well as the number of cells exhibiting at least five TIFs (Figure 2b–e). Ang‐(1‐7) was able to attenuate cell senescence and DNA damage triggered by IL‐1β, and once again, this effect was dampened by A779 (Figure 2a–e). As observed for Ang II, Ang‐(1‐7) was also capable to prevent the SA‐β‐gal+ staining induced by IL‐1β (1.16 ± 0.07‐fold over basal unstimulated staining) in late‐passage senescent cells (Supporting Information Figure S2b).

### 2.3 Ang‐(1‐7) attenuates the endothelial senescent pro‐inflammatory phenotype

Senescent endothelial cells undergo a series of changes that result into an activated pro‐inflammatory and secretory phenotype that eventually favors leukocyte recruitment.

**Figure 3:**
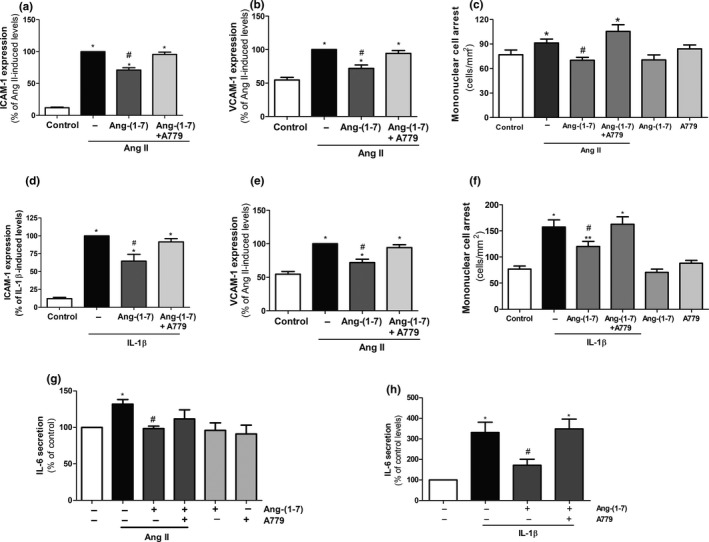
Ang‐(1‐7) attenuates the endothelial senescent pro‐inflammatory phenotype. The levels of (a, d) ICAM‐1 and (b, e) VCAM‐1 were assessed by flow cytometry in HUVEC cultures treated for 18 h with Ang II (100 nM) or IL‐1β (2.5 ng/ml), respectively, either alone or in the presence of Ang‐(1‐7) (100 nM). In some experiments, the Mas receptor antagonist A779 (1 μM) was added. (c, f) Adhesion of human mononuclear cells to HUVEC monolayers exposed to the treatments described above. (g, h) IL‐6 secretion in supernatants from HUVEC exposed to the treatments described above*. n* = 3–8. *p ≤ 0.05 vs. control untreated cells, ^#^ *p* ≤ 0.05 vs. Ang II‐ or IL‐1β‐stimulated cells

Consistent with this notion, both Ang II and IL‐1β enhanced the expression of the adhesion molecules, intercellular adhesion molecule (ICAM)‐1 (Figure 3a,d) and vascular cell adhesion molecule (VCAM)‐1 (Figure 3b,e), and used as markers of endothelial cell activation and SASP acquisition. Functionally, the endothelial activation by both Ang II and IL‐1β translated into high numbers of superfused mononuclear cells adhered to HUVEC monolayers (Figure 3c,f). Ang‐(1‐7) alleviated the pro‐adhesive actions of both Ang II and IL‐1β, and such effect was abolished in the presence of A779 (Figure 3a–f). Neither Ang‐(1‐7) nor A779 did influence by themselves cell adhesion (Figure 3c,f). Moreover, Ang II and IL‐1β enhanced the transcription (Supporting Information Figure S3a,b) and the secretion of the pro‐inflammatory cytokine IL‐6, a key SASP marker. Ang‐(1‐7) was able to inhibit the induction of IL‐6 secretion by both Ang II and IL‐1β, while A779 was in turn able to revert the anti‐inflammatory action of Ang‐(1‐7) over the SASP marker (Figure 3g,h).

### 2.4 Klotho is required for the anti‐senescence action of Ang‐(1‐7)

**Figure 4:**
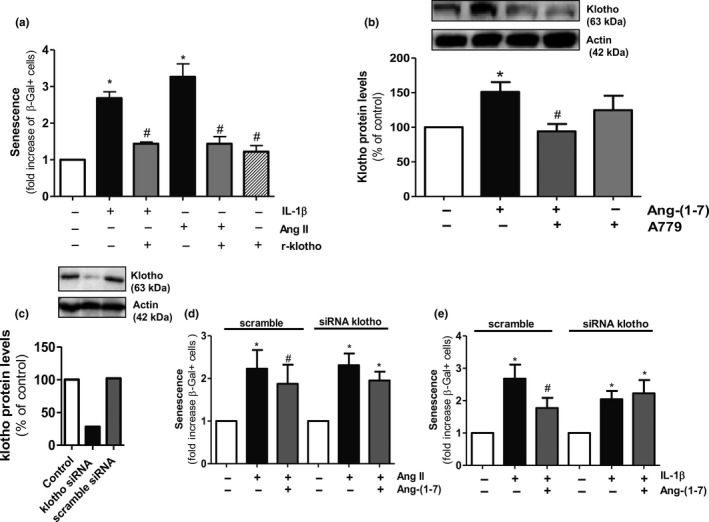
Klotho mediates the anti‐senescence effect of Ang‐(1‐7). (a) HUVEC were treated for 18 h with Ang II (100 nM) or IL‐1β (2.5 ng/ml), either alone or with r‐klotho (1 nM), and the number of SA‐β‐gal+ cells was determined. *n* = 3. (b) Klotho protein levels were measured by Western blot in HUVEC stimulated for 18 hr with Ang‐(1‐7) (100 nM) alone or in combination with A779 (1 μM). A representative gel is shown on top. *n* = 5–7. (c) Representative experiment showing klotho protein levels in HUVEC untreated or transfected with klotho siRNA or scramble siRNA (negative control). A gel is shown on top. SA‐β‐gal+ were determined in HUVEC transfected with scramble siRNA or klotho siRNA and treated for 18 hr with (d) Ang II (100 nM) or (e) IL‐1β (2.5 ng/ml), alone or in combination with Ang‐(1‐7) (100 nM). *n* = 4–5. *p ≤ 0.05 vs. control untreated cells, ^#^ *p* ≤ 0.05 vs. Ang II‐ or IL‐1β‐stimulated cells

To analyze the cytoprotective mechanisms that might be on the basis of the anti‐senescence action of Ang‐(1‐7), we first explored a possible role for klotho. In endothelial cells, human recombinant klotho (r‐klotho) prevented the enhanced SA‐β‐gal+ cell number induced by both Ang II and IL‐1β, without affecting cell senescence by itself (Figure 4a). In fact, Ang‐(1‐7) was itself capable to augment klotho levels in endothelial cells via Mas receptors (Figure 4b). To assess whether klotho was necessary for the anti‐senescence action of Ang‐(1‐7), endothelial cultures were next transfected with either klotho siRNA or scrambled sequence non‐silencing siRNA (Figure 4c). In klotho‐silenced cells, but not in those transfected with scrambled siRNA, the capacity of Ang‐(1‐7) to antagonize the senescence response triggered by both Ang II and IL‐1β was fully lost (Figure 4d,e).

### 2.5 Nrf2/HO‐1 mediates the anti‐senescence effect of Ang‐(1‐7) and klotho

**Figure 5:**
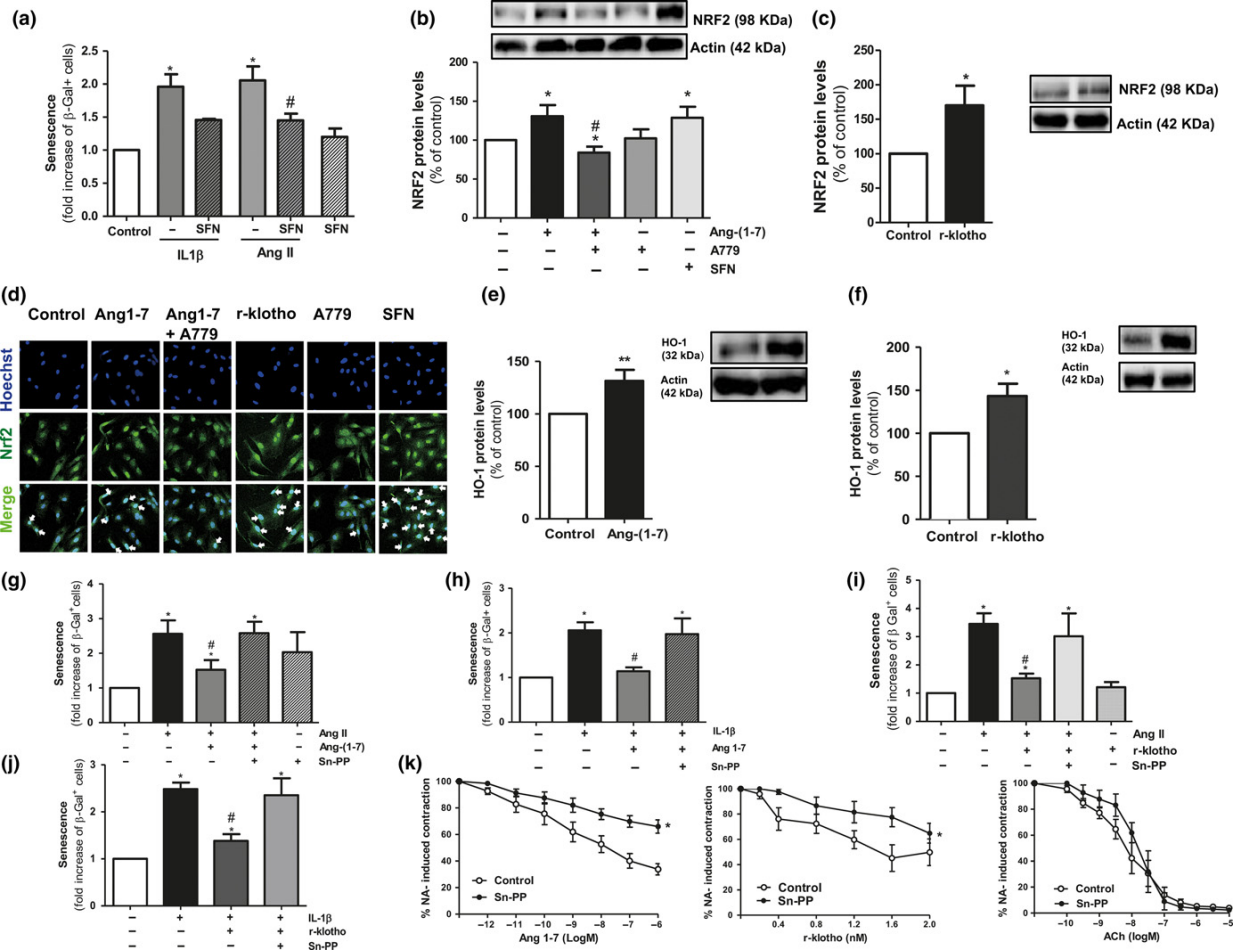
HO‐1 mediates the anti‐senescence effect of klotho and Ang‐(1‐7). (a) SA‐β‐gal+ cells were quantified in HUVEC cultures treated for 18 h with either Ang II (100 nM) or IL‐1β (2.5 ng/ml) alone or with the Nrf2 activator sulforaphane (SFN; 1 μM). *n* = 3. (b, c) Nrf2 protein levels were measured by Western blot in HUVEC stimulated for 18 hr with Ang 1‐7 (100 nM) alone or with A779 (1 μM). SFN (1 μM) was used as a positive control. Nrf2 was also quantified in HUVEC exposed to human r‐klotho (1 nM) for the same time period. *n* = 7–10. (d) Nrf2 (green) was visualized by indirect immunofluorescence in HUVEC stimulated for 18 hr with Ang‐(1‐7) (100 nM) alone or with A779 (1 μM), r‐klotho (1 nM), or SFN (1 μM). Nuclei counterstained with Hoechst (blue) (200×). (e, f) Determination of HO‐1 protein levels in HUVEC cultures treated for 18 hr with Ang (1‐7) (100 nM) or r‐klotho (1 nM). Representative blots are shown on the right. *n* = 5–15. (g, h) Quantification of SA‐β‐gal+cells in HUVEC cultures treated for 18 hr with either Ang II (100 nM) or IL‐1β (2.5 ng/ml) alone or in combination with Ang‐(1‐7) (100 nM) and/or tin protoporphyrin IX (Sn‐PP; 1 nM). *n* = 3–6. (i, j) SA‐β‐gal+cells were also quantified in cultures stimulated for 18 hr with either Ang II (100 nM) or IL‐1β (2.5 ng/ml) alone or in combination with r‐klotho with or without Sn‐PP (1 μM). *n* = 3–7. (k) Vasorelaxant responses of noradrenaline (NA; 3 µM) pre‐contracted murine microvessels to Ang‐(1‐7) (1 pM to 1 µM), r‐klotho (0.4 to 2 ng/ml), or acetylcholine (ACh; 0.1 nM to 10 μM). The relaxation curves were performed with or without (control) Sn‐PP (1 nM). *n* = 7–12 segments obtained from three to six animals. *p ≤ 0.05 vs. control untreated cells, ^#^ *p* ≤ 0.05 vs. Ang II‐ or IL‐1β‐stimulated cells

To gain insight into the common cytoprotective pathways that might be activated by both Ang‐(1‐7) and klotho, we next focused on Nfr2/HO‐1. First, the pharmacological activation of Nrf2 by means of sulforaphane protected endothelial cultures against cell senescence induced by both Ang II and IL‐1β (Figure 5a). Moreover, Ang‐(1‐7) augmented the cellular levels of Nrf2 in a Mas‐dependent manner (Figure 5b,d), as r‐klotho also did (Figure 5c,d).

HO‐1 levels were equally enhanced by either Ang‐(1‐7) or r‐klotho (Figure 5e,f). In fact, in cells exposed to the HO‐1 inhibitor tin protoporphyrin IX (Sn‐PP, 1 µM), the protective effect of Ang‐(1‐7) against both Ang II‐ and IL‐1β‐induced senescence was lost (Figure 5g,h). Sn‐PP also abrogated the anti‐senescence action of r‐klotho (Figure 5i,j). Similarly, the Nrf2 inhibitor trigonelline (1 µM) inhibited the anti‐senescence action of both Ang‐(1‐7) and r‐klotho in Ang II‐ or IL‐1β‐stimulated cells (Supporting Information Figure S4a, b). Moreover, in klotho‐silenced cells, Ang‐(1‐7) was not capable anymore to enhance Nrf2 levels and even a reduction in Nrf2 levels was observed (Supporting Information Figure S5).

Globally considered, these data identified Nrf2/HO‐1 as key mediators of the cytoprotective effects derived from the consecutive activation of Ang‐(1‐7) and klotho. This concept was further reinforced using a functional ex vivo vascular reactivity assay. In noradrenaline pre‐contracted murine mesenteric microvessels, the vasodilation exerted by Ang‐(1‐7), but also the one induced by r‐klotho, was markedly inhibited by Sn‐PP, while the one exerted by the vasodilator acetylcholine remained unaffected (Figure 5k).

## 3 DISCUSSION

Vascular aging is a main risk factor for developing adverse cardiovascular events (Laina, Stellos, & Stamatelopoulos, 2017). Moreover, in addition to physiological aging, certain pathological conditions, such as type 2 diabetes mellitus, obesity, or chronic kidney disease, display accelerated vascular aging, which aggravates the complications of the disease (Kooman, Kotanko, Schols, Shiels, & Stenvinkel, 2014; Nilsson, 2008). Overall, the global aging population together with the high prevalence of the above‐mentioned diseases underpins the necessity to identify therapeutic strategies to retard vascular aging and its adverse outcomes.

In the present study, we show that the RAS, and particularly the Ang‐(1‐7)/Mas axis, may be a suitable target to delay endothelial cell senescence, one of the key hallmarks of vascular aging that drives to endothelial dysfunction and atherosclerosis.

We herein demonstrate that Ang‐(1‐7) attenuates total and telomeric DNA damage, an early senescence‐associated event that signals downstream to provoke growth arrest and senescence (López‐Otín et al., 2013). Ang‐(1‐7) also mitigates SA‐β‐gal activity, which reflects the increased lysosomal mass observed in senescent cells (Kurz, Decary, Hong, & Erusalimsky, 2000), and attenuates the SASP that promotes leukocyte adhesion and inflammation. All these anti‐senescence actions of Ang‐(1‐7) relied on the activation of the Mas receptors, as for other vascular and non‐vascular protective actions of Ang‐(1‐7) (Machado‐Silva et al., 2016; Peiró et al., 2016; Villalobos et al., 2016). In line with our findings, a recent microarray‐based study has identified the senescence‐associated p53 signaling pathway as a main protein cluster influenced by Ang‐(1‐7) in endothelial cells (Meinert et al., 2016).

Besides Ang II, the present study shows that the pro‐inflammatory cytokine IL‐1β can also promote human endothelial cell senescence. The role of this cytokine in human cardiovascular disease and atherosclerosis has recently gained relevance at the light of clinical trials such as CANTOS (Libby, 2017; Ridker et al., 2017). Importantly, Ang‐(1‐7) was also capable to mitigate the endothelial senescence induced by IL‐1β. This suggests pleiotropic benefits of the Ang‐(1‐7)/Mas receptor axis beyond Ang II and the RAS, which reinforces the potential interest of the heptapeptide as a tool for fighting vascular disturbances. In late‐passage cultures, Ang‐(1‐7) maintained not only its capacity to attenuate the impact of extracellular stressors but also reduced to some extent the increased number of SA‐β‐gal+ cells observed in unstimulated conditions, suggesting that the heptapeptide may prove useful in counteracting, at least in part, replicative senescence.

Klotho is currently acknowledged as an anti‐aging protein, but its role in the vasculature still remains poorly understood. Similar to Ang‐(1‐7), klotho is able to exert nitric oxide‐dependent vasorelaxant actions (Saito et al., 2000, 1998). Klotho also protects human endothelial cells against the harmful effects of oxidative stress by activating superoxide dismutase and thus exerting anti‐apoptotic and anti‐senescence properties (Ikushima, Rakugi, & Ishikawa, 2006; Yamamoto et al., 2005). In human vessels, the expression of klotho positively correlates with the expression of the anti‐inflammatory cytokine IL‐10 (Martín‐Núñez et al., 2017). Clinically, circulating klotho deficiency has been proposed to be a biomarker of chronic kidney disease and vascular age‐related diseases (Hu et al., 2014), and soluble and vascular klotho inversely correlates with inflammatory markers in human atherosclerosis (Martín‐Núñez et al., 2017). Overall, the still limited available evidence supports a protective anti‐oxidant and anti‐inflammatory role of klotho in the vasculature. Here, we provide data that clearly reinforce the anti‐senescence and vasorelaxant properties attributed to klotho, but, most importantly, by using gene silencing approaches, we identify for the first time klotho as a fundamental mediator of the protective anti‐senescence actions of Ang‐(1‐7). To date, the regulation of klotho expression in endothelial cells remains poorly understood, and hence, the intracellular mechanisms by which Ang‐(1‐7) may induce klotho still need to be determined.

**Figure 6:**
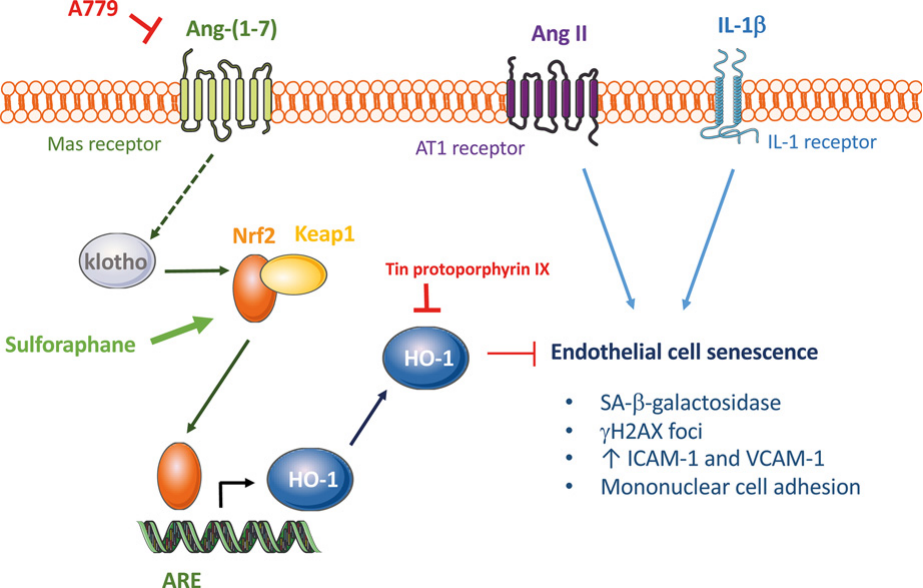
Graphical abstract depicting the proposed mechanism of action for the anti‐senescence properties of Ang‐(1‐7) in endothelial cells

Inflammation and oxidative stress are hallmarks of aging and age‐related pathologies (El Assar et al., 2012; López‐Otín et al., 2013; Rodríguez‐Mañas et al., 2009). In recent years, the Nrf2/HO‐1 system has been regarded not only as a modulator of aging and species longevity, but also as a promising therapeutic target in the vasculature to ameliorate pathologies such as restenosis, thrombosis, myocardial infarction, hypertension, or atherosclerosis (Durante, 2010). Here, we show that this powerful defense system is activated by Ang‐(1‐7) in human endothelial cells. In line with our observations, a non‐peptide Mas agonist drug AVE0991 was reported to activate HO‐1 expression in another vascular cell type such as vascular smooth muscle (Sheng‐Long et al., 2012). In fact, we demonstrate that the Nrf2/HO‐1 is necessary for klotho itself to protect against senescence, which is in line with very recent findings in another vascular cell type (Maltese et al., 2017). Moreover, we identify HO‐1 as a main player in the vasorelaxant actions of klotho and Ang‐(1‐7) in a more complex ex vivo system. Overall, the Nrf2/HO‐1 system arises as a fundamental common mediator of the vasculoprotective actions of klotho and Ang‐(1‐7) (Figure 6).

In conclusion, the present study reveals the RAS heptapeptide Ang‐(1‐7) as a valuable tool to protect against human endothelial cell senescence through the consecutive activation of klotho and the Nrf2/HO‐1 axis. Over the last years, a notable effort is being made to develop pharmacological activators of the ACE2/Ang‐(1‐7)/Mas axis to fight a number of conditions, including cardiovascular diseases. In light of the present results, these drugs may prove useful to counteract endothelial cell senescence and premature vascular aging, as well as their deleterious complications. Since blocking senescence with Ang‐(1‐7) might promote dysplasia or neoplasia of endothelial cells, or Ang‐(1‐7) may itself exert hypotension or cardiac and renal fibrosis (Shao et al., 2008; Velkoska, Dean, Griggs, Burchill, & Burrell, 2011), further clinical studies will be needed to assess not only the efficacy but also the safety and tolerability of the therapeutic use of Ang‐(1‐7).

## 4 EXPERIMENTAL PROCEDURES

### 4.1 Materials

Culture plasticware was from TPP (Trasadingen, Switzerland). M199 medium, fetal calf serum (FCS), and trypsin‐EDTA were from Biological Industries (Beit‐Haemek, Israel). Human r‐klotho and IL‐1β were purchased from Abcam (ab84072; Cambridge, UK) and Peprotech (London, UK), respectively. Ang‐(1‐7) and the Mas antagonist A779 were purchased from Bachem (Bubendorf, Switzerland). Sn‐PP and sulforaphane were from Frontier Scientific (Logan, UT, USA) and LKT Laboratories (Minnesota, USA), respectively. All other reagents were purchased from Sigma (St. Louis, MO, USA) unless otherwise stated.

### 4.2 Cell culture

Human umbilical vein endothelial cells (HUVEC) were isolated from umbilical cords, as previously described (Romacho et al., 2013). Cells were cultured in M199 medium supplemented with 20% FCS, 25 μg/ml endothelial cell growth supplement (ECGS), 100 μg/ml heparin, and antibiotics (100 U/ml penicillin, 100 µg/ml streptomycin, and 2.5 µg/ml amphotericin B) at 37°C in a humidified atmosphere with 5% CO_2_. For experiments, pre‐senescent cells at passages 1–5 were incubated for the indicated time periods with the different test compounds in M199 medium supplemented with 10% FCS, ECGS, heparin and antibiotics. For some experiments, senescent HUVEC were used at passage 12. All the procedures were reviewed and approved by the ethics committee of Universidad Autónoma of Madrid and Hospital Universitario La Paz, respectively, and written informed consent was obtained from all cord donors.

### 4.3 Determination of senescence‐associated β‐galactosidase (SA‐β‐gal) activity

SA‐β‐gal staining was performed with a commercial kit (Sigma, St. Louis, MO, USA) as previously described (Cardus, Uryga, Walters, & Erusalimsky, 2013; Villalobos et al., 2014). Positive senescent cells stained in blue were counted by blind observers under an Eclipse TE300 microscope (Nikon, Tokyo, Japan). The ratio of SA‐β‐gal+ cells per sample was determined by manual scoring of at least 1,000 cells counted in 12 randomized fields.

### 4.4 Detection of γH2AX foci and telomere dysfunction‐induced foci

DNA damage *foci *and telomere dysfunction‐induced *foci* (TIFs) were examined by immunofluorescence microscopy as previously described (Cardus et al., 2013; Villalobos et al., 2016). Telomeres were detected with an anti‐telomere repeat binding factor‐1 (TRF‐1) mouse monoclonal antibody (clone TRF‐78, dilution 1/1,000; Abcam) followed by a goat anti‐mouse IgG Alexa Fluor conjugate (dilution 1/1,000; Invitrogen, Paisley, UK). γH2AX was detected with a rabbit polyclonal antibody against a synthetic phosphopeptide detecting residues surrounding Ser139 of human histone H2A.X (dilution 1/100, Cell Signaling, Danvers, MA), followed by Alexa Fluor 594‐conjugated goat anti‐rabbit IgG (dilution 1/5,000, Invitrogen). Nuclei were counterstained with 4′,6′‐diamidino‐2‐phenylindole (DAPI; Invitrogen). After mounting, samples were viewed with a Nikon Eclipse 80i microscope. 40 to 50 Z‐stack fluorescence images were captured at 0.2‐mm intervals with a Hamamatsu Orca 285 digital camera, using the Volocity 3D image analysis software (Perkin Elmers, Inc., version 5.5). High‐resolution images were deconvolved using the Volocity Restoration module. To determine co‐localization in three dimensions, Z‐stacks were converted to voxels (volume pixels) and further analyzed with the Volocity Co‐localization module after image thresholding. The average green, red, and co‐localized fluorescence (expressed as voxels per cell) and the percentage of TIF‐positive cells (cells with five or more sites of co‐localization) were determined by analyzing at least 200 nuclei in 10 randomly selected fields per treatment.

### 4.5 Flow cytometry

The expression of VCAM‐1 and ICAM‐1 was measured by flow cytometry, as previously described (Azcutia et al., 2010). Primary antibodies against VCAM‐1 (clone IE5; Chemicon, Temecula, CA) or ICAM‐1 (clone 6.5B5; Chemicon) were used at a 1/100 dilution, followed by incubation with an appropriate Alexa Fluor 488 secondary antibody (Molecular Probes, Invitrogen Corporation, Carlsbad, CA; dilution 1/250). Fluorescence was measured in a FACScan flow cytometer (Beckton‐Dickinson, Franklin Lakes, NJ), and data were analyzed using CXP analysis software (Beckton‐Dickinson).

### 4.6 Adhesion assay

Mononuclear cells were obtained from buffy coats of healthy donors by Ficoll‐Hypaque density gradient centrifugation, as previously described (Mateo et al., 2007) following the principles outlined in the Declaration of Helsinki and the procedure was approved by the institutional ethics committee of the University Clinic Hospital of Valencia, Valencia, Spain. All subjects had signed an informed consent. Adhesion of mononuclear cells to HUVEC monolayers was analyzed with a live imaging flow model as previously described (Azcutia et al., 2010). Briefly, HUVEC monolayers were exposed for 18 hr to the different compounds. The Glycotech flow chamber was assembled and placed on an inverted microscope stage, and freshly isolated mononuclear cells (1 × 10^6^/ml) were then perfused across the endothelial monolayer. In all experiments, leukocyte interactions were determined after 5 min at 0.5 dyn/cm^2^. Cells interacting with the surface of the endothelium were visualized and recorded (×20 objective, ×10 eyepiece) using phase‐contrast microscopy (Axio Observer A1 Carl Zeiss microscope, Thornwood, NY).

### 4.7 IL‐6 secretion

After the appropriate treatments and incubation times, supernatants were collected, centrifuged at 900 *g* for 10 min at 4°C, and frozen at −20ºC until further use. IL‐6 was measured with an ELISA immunoassay (Raybiotech, Norcross, GA, USA) according to the manufacturer instructions.

### 4.8 Western Blot

Klotho, Nrf2, and HO‐1 levels in HUVEC were determined by Western blot as previously described (Romacho et al., 2013) using antibodies against klotho (ab203576; Abcam, Cambridge, UK; 1/1,000), Nrf2 (H‐300, SC‐13032, Santa Cruz Biotechnology, 1/1,000), HO‐1 (ab13243; Abcam; 1/10,000), or anti α‐actin primary antibody (dilution 1/50.000; Sigma‐Aldrich) to ensure equal loading, followed by incubation with corresponding specific horseradish peroxidase‐conjugated secondary antibodies (Bio‐Rad; 1:10,000). Immunoreactive bands were detected using an ECL detection kit (GE Healthcare) and quantified by densitometry using the NIH software Image J.

### 4.9 Indirect immunofluorescence

Nrf2 was visualized in HUVEC by indirect immunofluorescence as previously described (Villalobos et al., 2016). A primary polyclonal antibody against Nrf2 (dilution 1/500; H‐300, SC‐13032, Santa Cruz Biotechnology) was used, followed by incubation with an appropriate secondary antibody (dilution 1/800). Nuclei were counterstained with Hoechst (5 µg/ml, Invitrogen), and cells were observed with a confocal microscopy (TCS SPE, Leica, Wetzlar, Germany).

### 4.10 α‐Klotho siRNA transfection

Cells were seeded in M199 culture medium with 20% FCS without antibiotics for 24 hr, and klotho was silenced by adapting a previously described method (Peiró et al., 2013). Small interfering RNA (siRNA) encoding human klotho (Thermo Fisher; 50 nM) was transfected using Lipofectamine RNAiMAX and Opti‐MEM Reduced Serum Medium (Thermo Fisher). After 6.5 hr, Opti‐MEM was replaced by M199 20% FCS without antibiotics. Cells were exposed to the different treatments in M199 with 10% FCS without antibiotics for 24 hr and then accordingly processed. klotho siRNA duplexes sequence is as follows: 5′‐GGA UGU CCA CCA CAG UAA ATT‐3′ and 5′‐UUU ACU GUG GUG GAC AUC CCA‐3′. A scrambled duplex of RNA not targeted to any human gene was used as a negative control.

### 4.11 Microvascular reactivity

Four‐month‐old male C57Bl/6 mice were maintained under standardized conditions with an artificial 12‐hr–12‐hr dark–light cycle, with free access to food and water. All animal studies followed national guidelines and were approved by the institutional animal care and ethics committees. Mice were sacrificed by exposition to carbon dioxide. Rings of first branch mesenteric arteries (internal diameter: 150–200 µm) were mounted on a small‐vessel myograph to measure isometric tension as described before (Peiró et al., 2016). Arteries were contracted with 3 µM noradrenaline, and then, the vasoactive responses to Ang‐(1‐7) (1 pM to 1 µM), acetylcholine (0.1 nM to 10 µM), or klotho (r‐klotho; 0.4–2 nM) were tested. In some cases, the mesenteric segments were preincubated for 20 min with Sn‐PP (1 µM) before addition of noradrenaline.

### 4.12 Statistical analysis

Results are expressed as mean ± SEM. Statistical analysis was performed using Student's *t* test and one‐way ANOVA followed by Bonferroni post hoc test or two‐way ANOVA, as appropriate. A *p *value ≤0.05 was considered statistically significant.

## CONFLICT OF INTEREST

None declared.

## AUTHOR'S CONTRIBUTIONS

C.P., C.F.S.‐F., and T.R. conceived the study. A.S.‐H.L., A.R., L.A.V., S.V., I.V., P.M. R.L., N.P.‐L., and I.S.‐P. performed in vitro and ex vivo experiments, analyzed raw data, performed statistical analysis, and drew figures. J.D.E. and M.J.S. designed and analyzed the DNA damage foci and flow chamber experiments, respectively. C.P., C.F.S.‐F., and T.R. wrote the manuscript. J.L.B., J.D.E., and M.J.S. contributed to the discussion. All authors reviewed and approved the final version of the manuscript.
