# Supplementary material for: The angiotensin‐(1‐7)/Mas receptor axis protects from endothelial cell senescence via klotho and Nrf2 activation
Source: Aging Cell. 2019 Feb 17;18(3):e12913. doi: 10.1111/acel.12913 (PMC6516147; doi:10.1111/acel.12913)

ONLINE DATA SUPPLEMENT

THE ANGIOTENSIN-(1-7)/MAS RECEPTOR AXIS PROTECTS FROM ENDOTHELIAL CELL SENESCENCE VIA KLOTHO AND NRF2 ACTIVATION

by

Alejandra Romero*, Álvaro San Hipólito-Luengo*, Laura A. Villalobos, Susana Vallejo, Inés Valencia, Patrycja Michalska, Natalia Pajuelo-Lozano, Isabel Sánchez-Pérez, Rafael León, José Luis Bartha, María Jesús Sanz, Jorge D. Erusalimsky, Carlos F. Sánchez-Ferrer , Tania Romacho**, Concepción Peiró**

*Both authors contributed equally to this work

**These authors jointly directed this work

Gene expression analysis of IL-6

Total RNA was extracted with an RNeasy kit (Qiagen, Courtaboeuf, France) following the manufacturer's instructions. The gene expression levels of the pro-inflammatory cytokine IL-6, was assessed by RT-qPCR using a ABI7500 cycler. Data were expressed as the ratio to a house-keeping gene (GAPDH). Primer sequences are given on Table 1.

Staining for senescence-associated β-galactosidase (SA- β-gal)

SA-β-gal staining was assessed as previously described in the Methods section in the manuscript.

Cell proliferation assay

For proliferation assays, pre-senescent (passage 3) and senescent (passage 12) HUVECs were seeded on 24-well plates at a density of 15,000 cells/well in M199 containing 20% fetal calf serum and allowed to attach for 3 h. Medium was then exchanged for 1% fetal calf serum-containing medium. Cells were allowed to proliferate for 48 h or 96 h with daily exchanges of fresh medium and treatments. After this period, cells were fixed with 1% glutaraldehyde, washed with phosphate-buffered saline (PBS) and stained with 1% crystal violet (Sigma-Aldrich). After washing with deionized water and drying, crystal violet was extracted with 10% acetic acid and proliferation was determined by reading absorbance at 595 nm, as previously described (Angulo et al., 2011).

References

Angulo J, Peiró C, Romacho T, Fernández A, Cuevas B, González-Corrochano R…Cuevas P. (2011) Inhibition of vascular endothelial growth factor (VEGF)-induced endothelial pro-liferation, arterial relaxation, vascular permeability and angiogenesis by dobesilate. European Journal of Pharmacology. 667(1-3):153-9. https://doi.org/10.1016/j.ejphar.2011.06.015.

Table1 : Real-Time PCR Primer Sequence

Human gene	Forward	Reverse
**IL6**	GCCAGAGCTGTGCAGATGAG	CAGTGGACAGGTTTCTGACC
**GAPDH**	GAGAGACCCTCACTGCTG	GATGGTACATGACAAGGTGG

Figure Legends

Figure S1: Replicative senescence reduces proliferation rate in HUVEC.

Proliferation rate was assessed by crystal violet staining in pre-senescent HUVEC (passage 3) and senescent HUVEC (passage 12). *n*=4 independent experiments. *p<0.05 vs. pre-senescent cells at corresponding time point. Representative SA-β-gal+ staining microphotographs (40X) from basal unstimulated pre-senescent HUVEC (passage 3) and senescent HUVEC (passage 12) are shown on top.

Figure S2: **Ang-(1-7) prevents the effect of IL-1β and Ang II in late passage senescent cells.** SA-β-gal+ cells were quantified in senescent HUVEC (passage 12) treated for 18 h with IL-1β (2.5 ng/ml), either alone or in the presence of Ang-(1-7) (100 nM) with or without the Mas receptor antagonist A779 (1 μM). *n*= 3 independent experiments. *p<0.05 vs. control untreated cells, #p<0.05 vs. IL-1β or Ang II.

Figure S3: Ang II and IL-1β and induce the gene expression of the SASP marker IL-6.

HUVECs were treated for 18 h with Ang-(1-7) (100 nM) or IL-1β (2.5 ng/ml). IL-6 mRNA levels were measured by qRT-PCR. *n*=4 independent experiments *p<0.05 vs. corresponding control (untreated cells).

Figure S4: Nrf2 mediates Ang-(1-7) and klotho anti-senescence effect in IL-1β and Ang II-stimulated HUVECs. SA-β-gal+ cells were quantified in HUVECs treated for 18 h with with IL-1β (2.5 ng/ml) or Ang II (100 nM), some cells were co-incubated with Ang-(1-7) (100 nM) or recombinant klotho (r-klotho; 1 nM) alone or in the presence of the Nrf2 inhibitor trigonelline (1 µM). *n*=3 independent experiments; $^{*}p<0.05$ vs. control (untreated cells), $^{\#}p<0.05$ vs. Ang-(1-7) treated cells in the presence of IL-1β or Ang II, respectively.

Figure S5: Klotho silencing prevents NRF2 induction by Ang-(1-7). HUVECs transfected with scramble siRNA or klotho siRNA (50 nM) were treated for 18 h with Ang-(1-7) (100 nM). NRF2 protein levels were determined by Western blot. *n*=3 independent experiments $^{*}p<0.05$ vs corresponding control (untreated cells), $^{\#}p<0.05$ vs. scramble untreated cells (control). A representative blot is shown on top.

Supplementary-1 (S1)

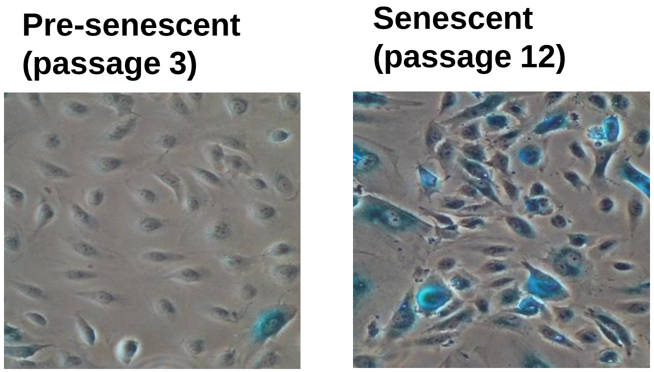

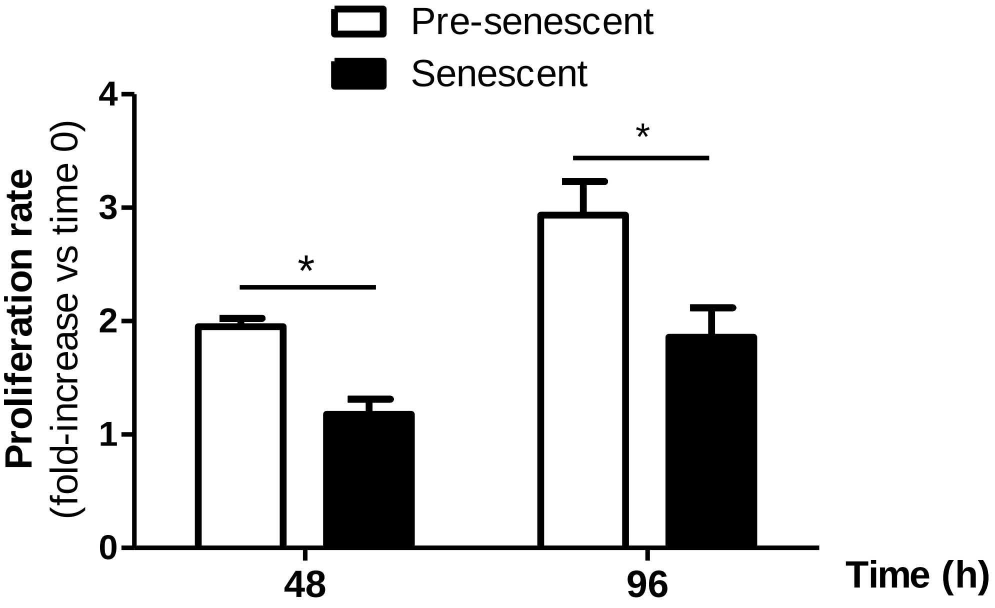

Supplementary-2 (S2)

A

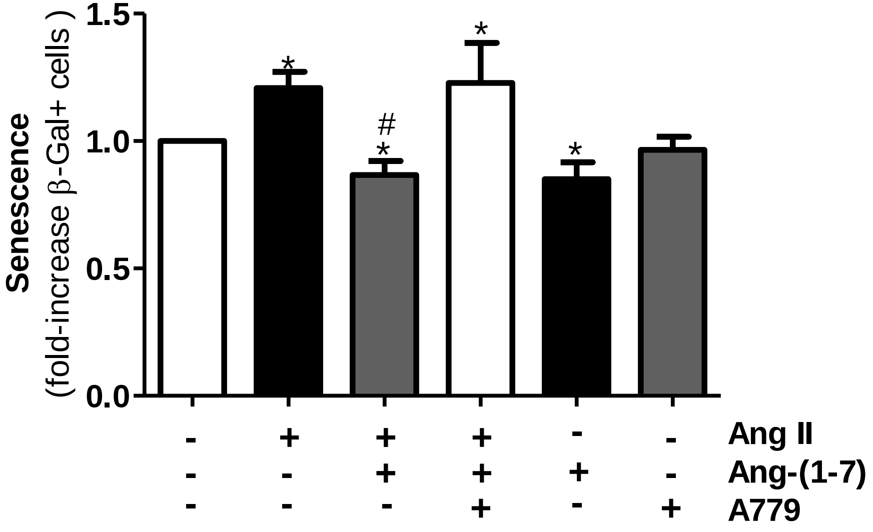

B

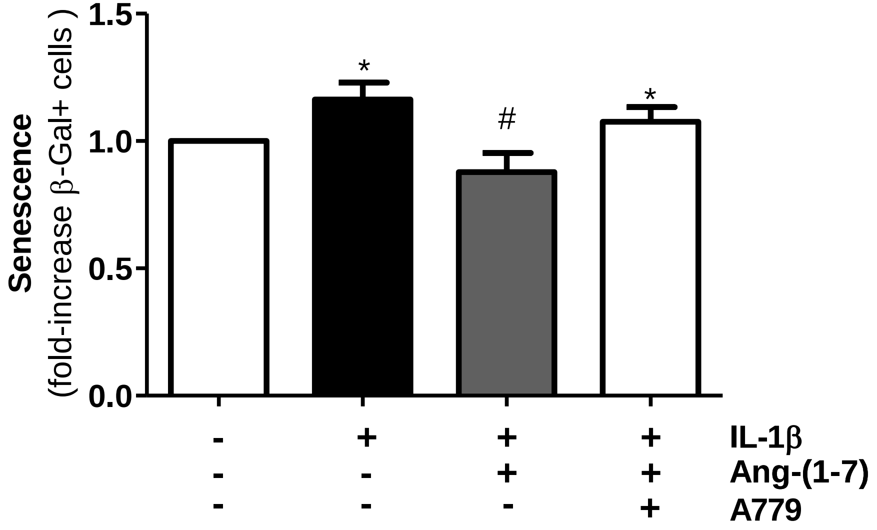

Supplementary-3 (S3)

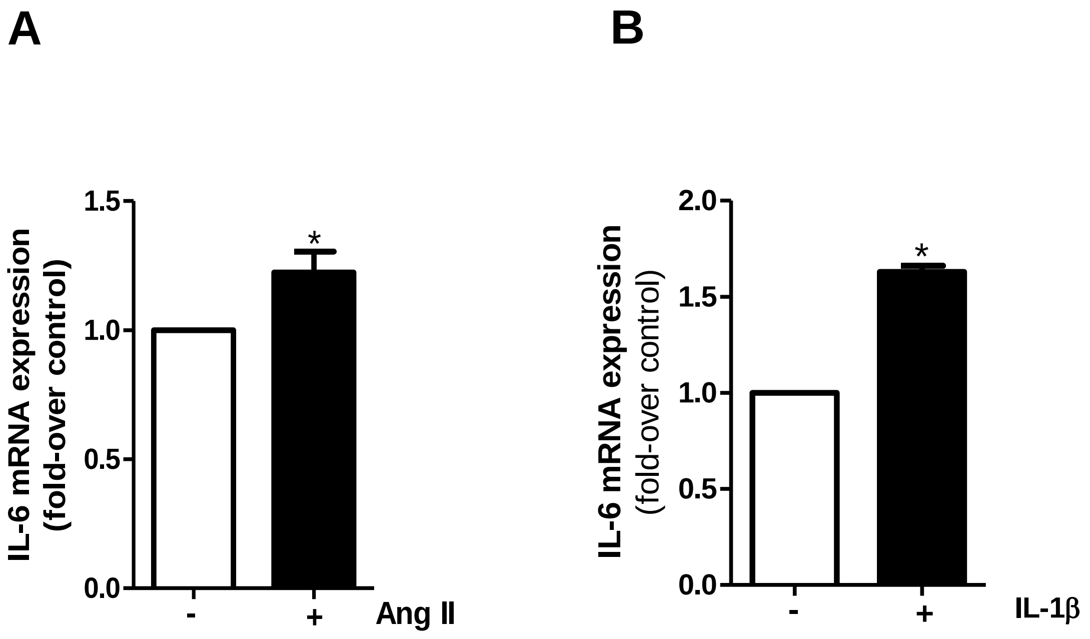

Supplementary-4 (S4)

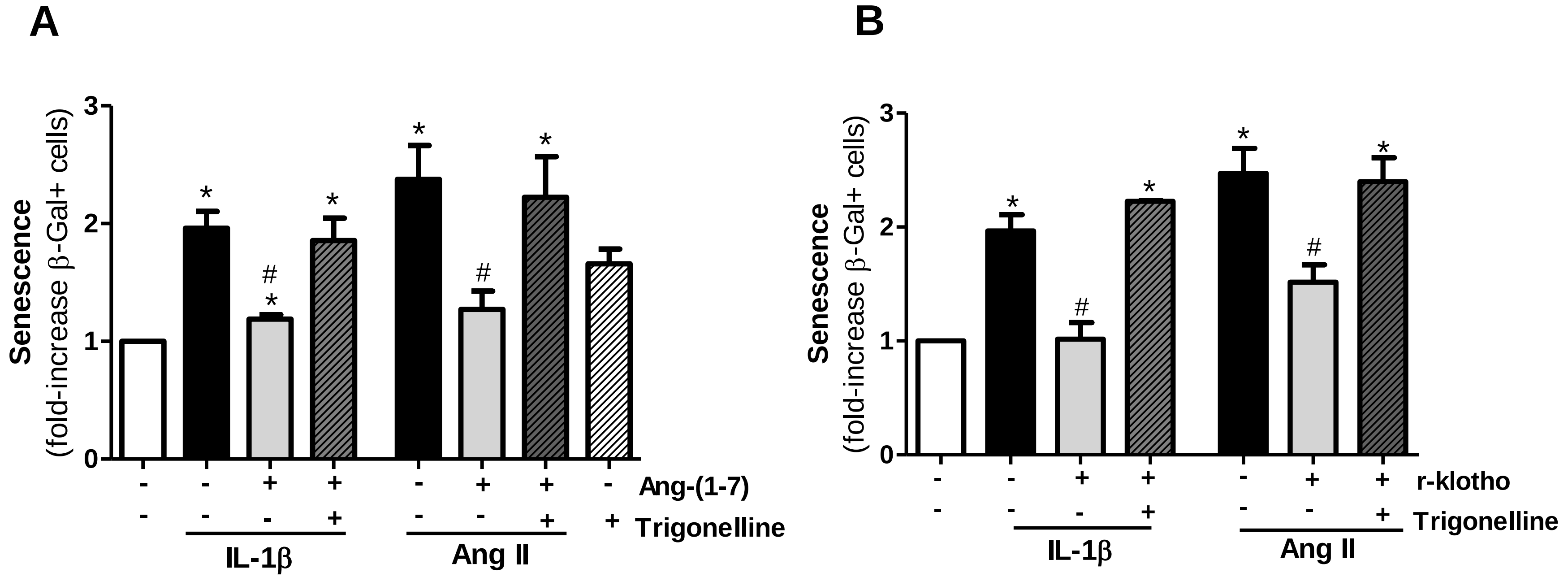

Supplementary-5 (S5)

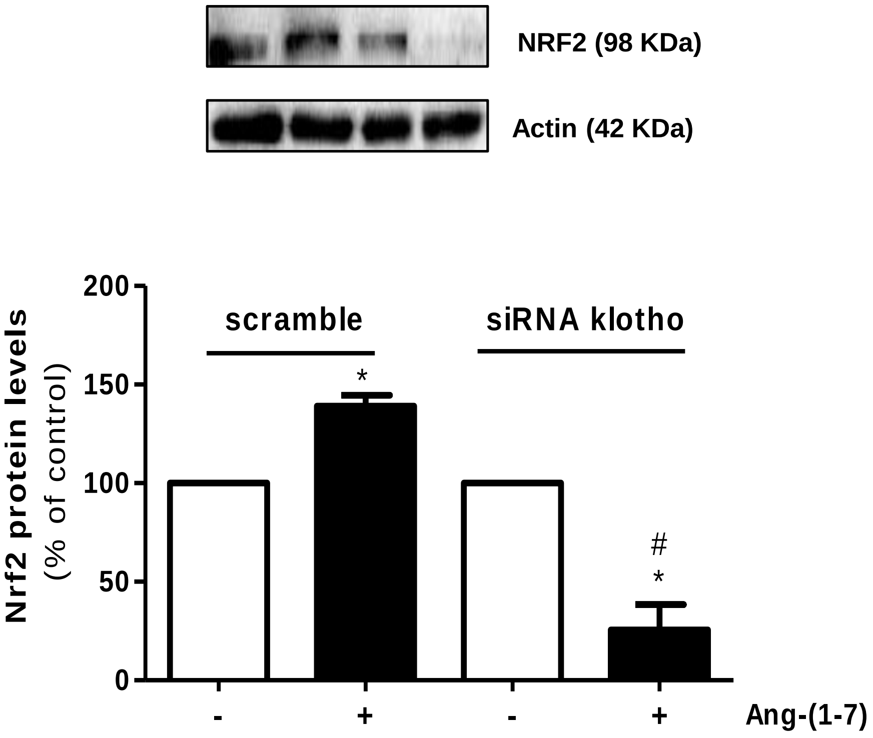

Supplement: Supplementary file 1 [file ACEL-18-e12913-s001.pdf]
